# A meta-analysis of amnion membrane in gingival recession

**DOI:** 10.6026/97320630019670

**Published:** 2023-05-31

**Authors:** Mohamed Lubaib, Shivani Dhawan, Ena Sharma, S.M Sivaraman, Tajinder Pal Singh Sandhu, Noel George

**Affiliations:** 1 Maharishi Markandeshwar College of Dental Sciences and Research (MMCDSR) Ambala, Haryana India; 2 Department of Epidemology and Biostatistics, KLE Academy of higher Education and Research, Karnataka India

**Keywords:** Recession, coronally advanced flap, amnion membrane, meta-analysis, recession depth

## Abstract

This systematic review was conducted to evaluate the effects of Amniotic Membrane (AM) as compared with other treatment modalities on the clinical outcomes, in gingival recession defects. Only Randomized controlled clinical trials published before 2020 were included. Studies were divided into 5 subgroups (1) Coronally advanced flap (CAF)+AM v/s Chorion membrane (CM) (2) CAF+AM v/s CAF+PRF (3) CAF+AM v/s CAF+Collagen membrane (4) CAF+AM v/s CAF (5) CAF+AM v/s CAF+ Subepithelial connective tissue graft (SCTG). Studies were evaluated for Recession Depth (RD) (Primary outcome); Clinical Attachment Level (CAL), Recession Width (RW) and Width of Keratinized Gingiva (WKG) (Secondary outcomes). The inverse variance approach was utilised in fixed or random effect models for the meta-analysis, which were chosen based on heterogeneity. Results suggested that the use of AM membrane showed comparable results in improving RD, RW, or CAL in the treatment of Miller Class-I and Class-II gingival recession compared to the other treatment modalities. However, CAF+AM resulted in statistically significant improvement in RD and RW than CAF+SCTG, though CAL gain was statistically more with CAF+SCTG. However, increase of WKG was found to be statistically significantly more in all the other treatment modalities as compared to CAF+AM. With properties like self-adherence, bioavailability and presence of growth factors AM with CAF can produce good aesthetic root coverage comparable to SCTG and PRF, where width of keratinized gingiva is adequate.

## Background:

Gingival recession is an intriguing and complex phenomenon. Literature has thoroughly documented that gingival recession defects can be successfully treated by several surgical approaches. The major goal is to improve the aesthetic appearance of the tooth by covering the exposed root. [1] However, there are some other objectives such as stopping the progression of active recession, increase the width of attached gingiva and reducing or eliminating dental hypersensitivity. Several techniques such as the free gingival graft (FGG), laterally or coronally positioned flaps (CAF), subepithelial connective tissue graft (SCTG), guided tissue regeneration (GTR) based root coverage procedures have been suggested to resolve the above-mentioned issues. [2] CAF is recommended surgical technique where there is presence of adequate keratinized gingiva apical to recession defect. The subepithelial connective tissue graft (SCTG) with coronally advanced flap (CAF) is considered as the gold standard technique for root coverage. [3] It offers better colour match and greater increase in the zone of an attached gingiva compared with other surgical techniques. However, disadvantage is the morbidity associated with the second surgical site required to harvest the autogenous palatal donor tissue. This can be overcome, by using recent advanced membranes as guided tissue regeneration techniques. The low predictability of regeneration is one of the fundamental flaws of the second-generation GTR membranes (collagen membrane). The stimulation of precursor cells with essential messenger molecules is required for predictable tissue regeneration. Third generation membranes, which operate as both barriers and delivery devices to release specific chemicals such as antibiotics, growth factors, and adhesion factors at the wound site and direct natural wound healing, have emerged, as the notion of tissue engineering has progressed. Amnion membrane is an example of third generation membrane. It contains specialized proteins such as fibronectin, laminin, proteoglycans, collagen type IV, V, and VII and various growth factors. It reduces inflammation, scar formation and act as natural biological barrier. [4, 5] Amnion membrane (AM) is one such biomaterial that has been used extensively for periodontal regeneration in recession defects. [6, 7] Therefore, it is of interest to document the systematic review and meta-analysis was to analyse the current evidence regarding use of AM with CAF in treatment of Class I and Class II gingival recession defects as compared to other treatment options.

##  Material and Methods:

## Protocol and registration:

The methodology of present systemic review followed the recommendations of the Cochrane Handbook for Systematic Reviews of Interventions. In order to increase the quality and research transparency, the methodology adhered to the Preferred Reporting Items for Systematic Reviews and Meta-Analyses (PRISMA) guideline checklists. [9] This review was registered in PROSPERO under the number CRD42021229436.

## Focused question (based on PICO strategy):

In the patients with Miller's class I and II gingival recession [Patient (P)], Is the use of Amnion Membrane [Intervention (I)] beneficial as compared to other treatment modalities [Comparison (C)], in terms of clinical outcome [Outcome(O)]? [10]

## Outcome measures:

Recession Depth (RD): measured from cemento-enamel junction (CEJ) to the gingival margin. Clinical Attachment Level (CAL): distance from a lower/ apical limit of the occlusal stent to the bottom of the pocket. Recession Width (RW): measured at the level of mid-buccal cement-enamel junction, keeping the probe horizontal and measuring the mesiodistal distance between the marginal gingiva. Width of Keratinized Gingiva (WKG): measured from mucogingival junction to the most coronal margin of the free gingiva, at the mid-buccal region.

## Search strategy:

All identified references from PubMed/MEDLINE, Cochrane Central Register of Controlled Trials, Web of Science, Wiley Online Library, Scopus, EBSCOHOST, and EMBASE databases were screened to include only human studies in English language. References of the included studies (cross referencing) were searched to obtain new studies by using MeSH terms, key words like "gingival recession", "periodontal plastic surgery", "mucogingival surgery", "root coverage procedure", Class I and II gingival recession", "amnion membrane", "chorion membrane", "placental membrane", "plastic surgery". Studies evaluating therapeutic use of amnion membrane with coronally advanced flap (CAF) alone or in combination with other biomaterials in Class I and II gingival recession defects from January 1, 2013 to December 31, 2020 were searched. Randomised controlled trails (RCTs) and prospective controlled trials were selected having follow-up period of ≥6 months. Animal studies, retrospective cohort studies, in vitro studies, case series, case reports, and reviews were excluded. All the authors conducted the search and screening process. Titles of studies with their abstracts were first carefully analysed, followed by selection of complete articles for thorough reviewing according to inclusion criteria for future data extraction.

## Data extraction:

**Figure 1:**
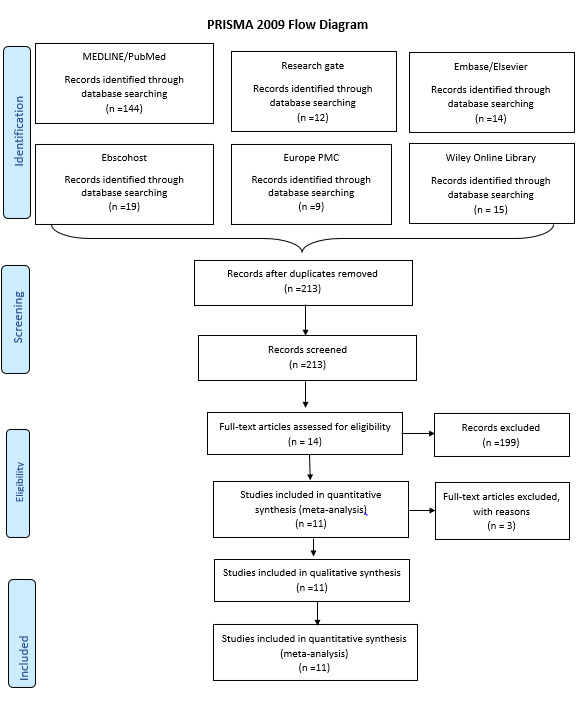
Flow diagram (PRISMA format) of the screening and selection process

The following data was extracted from the included studies: Authors, Study Design, Follow-up period, Number of treated Recession defect sites, Number of patients, Age and Gender of the patient, Miller Class types, Site of Recession defects, Surgical Technique, intervention and control group, RD and other outcomes between baseline and 6 months. The initial search yielded 213 publications: 144 MEDLINE/ PubMed titles, 14 Embase/Elsevier articles, 12 Research Gate articles, 15 Wiley Online Library, 9 Europe PMC, and 19 Ebscohost articles. 199 articles were eliminated after the initial assessment (title and abstract evaluation). Of the 14 potential articles, three studies were excluded. The reasons for the exclusion of potential studies were: in one study microsurgical technique was used [11] ; in another, the statistical analysis showed negative values [12]; and in the third study, follow up period was only 3 months [13]. Consequently, 11 RCTs [14,15,16, 17,18,19, 20,21,22, 23,24] published between 2013 and 2020 were included (Figure 1). The two [16,17] studies were conducted in parallel design and nine [14,15, 18-24] were conducted in split-mouth design. The trials had a total of 10 [15] to 51 [21] participants, ranging in age from 18 to 55 years. A total of 421 gingival recession defects (217 tests and 204 controls) were treated. Except in two investigations [18,21] in which amnion membrane was placed solely in Miller class I recession defects, amnion membrane was placed in Miller class I or II recession defects in all the remaining studies. Three researches [18, 21, and 23] clearly included both the maxilla and the mandible, one study only included the maxilla [16], while the remaining papers did not specify the sites. Three researches [16, 21, 23] included anterior teeth and premolars; one study [18] included canines; and the remaining eight studies had not mentioned any areas. Nine studies [14,15, 16,17,18, 19,20,22, 23,24] had a 6-month follow-up period, one had a 9-month follow-up period [16], and another [21] had a 5-year follow-up period (Table 1).

## Statistical analysis:

The continuous variables RD, CAL, RW and WKG of the included studies were categorized in groups and subgroups and analyzed using stata software. Mean difference (MD) or standardized mean difference (SMD) was used to estimate the effect, with 95% confidence intervals. Meta-analysis was performed using the random-effects model for the outcome. Heterogeneity was assessed with the X2 test, and the potential effect on meta-analysis was quantified with I2. Values up to 25% were classified as low heterogeneity, and values up to 50% or 70% were classified as medium or high heterogeneity respectively. When significant heterogeneity was observed (p<0.10) results of the random effects model were validated. When low heterogeneity was found, the results of the fixed effects model were considered. The level of statistical significance was set at p<0.05. Publication bias was explored graphically with funnel plots. Asymmetry in the funnel plots (studies outside the triangular area) indicated potential publication bias.

## Results:

**Figure 2:**
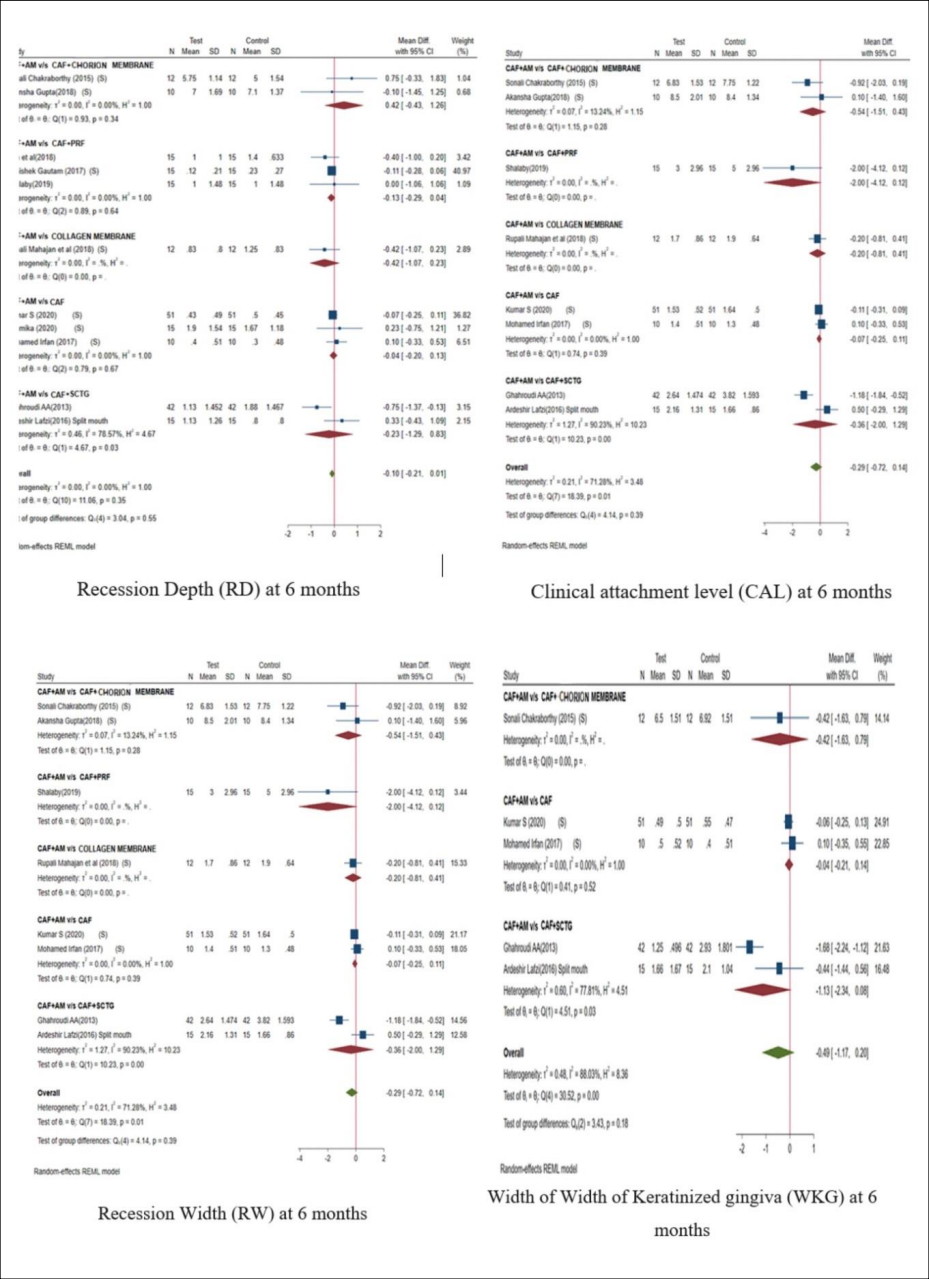
showing forest plot of RD, CAL, WKG and RW at 6 month

**Figure 3:**
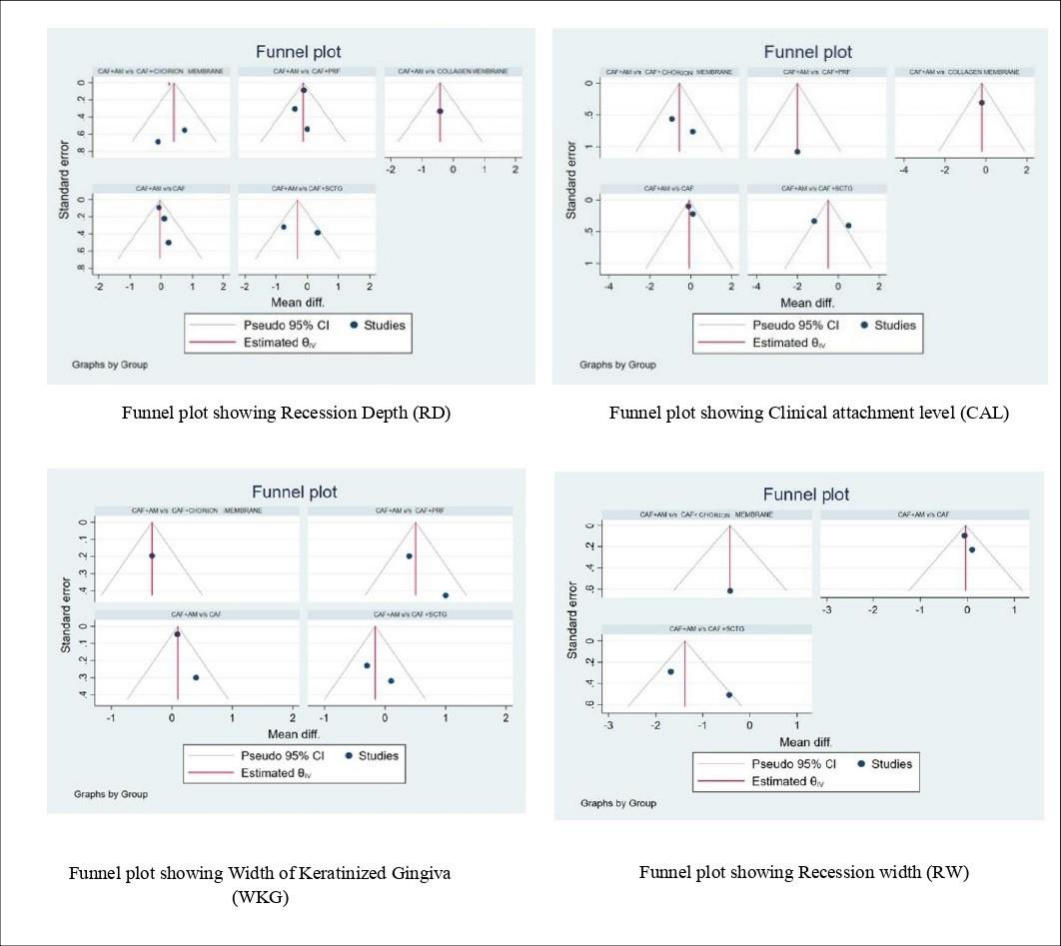
showing Funnel plot of RD, CAL, WKG and RW at 6 month

In case of Recession depth (RD), CAF+AM v/s CAF+SCTG showed statistically significant difference (p=0.03) in favour of test group (CAF+AM). The overall comparison results between test group and control groups did not show any statistically significant difference (p=0.55) (Figure 2). Regarding Clinical attachment level (CAL), CAF+AM v/s CAF+SCTG showed statistically significant difference (p=0.00) in favour of control group (CAF+SCTG). The overall comparison results at 6 months between test group and control groups did not show any statistically significant difference with p=0.39 (Figure 2). In case of Recession width (RW), CAF+AM v/s CAF+SCTG showed statistically significant difference with p=0.03 in favour of test group. The overall comparison results at 6 months between test group and control groups did not show any statistically significant difference with p=0.18 (Figure 2). In case of Width of keratinized gingiva (WKG), comparison of CAF+AM did not show any improvement over other control groups. The overall comparison results at 6 months between test group and control groups showed a statistical significance difference with p=0.03 in favour of control groups (Figure 2). The funnel plots did not indicate any asymmetric distribution in all parameters, which showed no possible publication bias. All the studies were present inside the triangular area of the 95% CI region (Figure 3).

## Discussion:

To the best of our knowledge, this is the first systematic review and meta-analysis investigating the effectiveness of AM for recession coverage in randomized controlled clinical studies compared with all other treatment modalities. Placental-based AM has inherent biologic properties that actively promote wound healing in lieu of simply providing an occlusive barrier for selective cell repopulation. AM not only maintains the structural and anatomical configuration of regenerated tissue, but also contribute to the enhancement of healing by providing rich source of stem cells and reduction of post-operative scarring and subsequent loss of function. It contains growth factors that aid in formation of granulation tissue by stimulating fibroblast growth and neovascularization. This meta-analysis searched for scientific evidence of effects of use of amnion membrane in Miller's class I and class II recession defects. Two studies [14,15] investigated the use of CAF+AM vs CAF+CM. No statistically significant advantage was observed between two groups for all parameters. Type I, IV, V, VI collagen, proteoglycans, laminin, and fibronectin are abundant in the collagen layers of amnion and chorion. Both the membranes have antibacterial and antimicrobial capabilities, and the presence of natural inhibitors of matrix metalloproteinases-1, 2, 3, 4, interleukin-10, and interleukin-1 receptor antagonists reduces inflammation at the wound site, causing reduction of Interleukin 1α and 1β. Compared to other membranes, the thickness of these membranes is less; but the added advantage of this membrane is self-adhering properties that aids in stabilization of this membrane without suturing [15] (Table 2).

Three studies [16-18] investigated the use of CAF+AM vs CAF+PRF. No statistically significant advantage was observed between two groups for all parameters compared with the CAF+PRF group. Because of drawbacks of PRF, such as the need for blood removal, expensive equipment, and longer treatment times; allografts such as amnion membrane has gained popularity. Low cost and convenient availability of AM make it a viable alternative to PRF and other autografts for both patients and operators (Table 2). There was only one study by Mahajan [19] that has compared the effect of CAF + AM vs CAF+ Collagen membrane. Significant improvements were observed in RD reduction, gain in CAL, and increase in gingival biotype in both groups from baseline to 6 months. However, intergroup comparison of these parameters yielded non-significant differences. AM has better handling properties than collagen because of its thickness, which makes it easier to manipulate. The ability of amnion allograft to self-adhere eliminates the need for sutures, making the procedure less technically demanding and reducing surgical time. It is a good choice for recession coverage in difficult to reach areas like the molar region (Table 2).

Three studies [20-22] investigated the use of CAF+AM vs CAF alone. The addition of AM to the CAF showed no statistically significant improvement in all parameters when it compared to CAF alone. This might be because interposing AM between an avascular surface (tooth) and flap was not favourable for complete root coverage. Also, AM undergoes some shrinkage with time, thus causing dead space between root surface and tissues that might invite microorganism and hampers healing. However, in studies by Irfan M [20] and Kumar S [21], thickness of keratinized gingiva with AM + CAF was more than CAF alone. This was because AM showed soft tissue augmentation from proliferation of gingiva and periodontal ligament fibroblast. Increase in the gingival thickness affects the long-term treatment outcomes because of difference in amount of blood supply to underlying bone and susceptibility to resorption. This was depicted from the fact that in study by Kumar S [21], who found that though comparable reduction is found between CAF with AM vs CAF alone with respect to RD, RW, CAL, and WKG at 6 months, but at the end of 5 years, the gain in all the parameters were found to be maintained in CAF with AM group as compared to CAF alone. This showed the role of AM in long-term benefit of reoccurrence of recession than short term (6 months) results. This might be because of thick gingival phenotype obtained by amniotic membrane (Table 2).

Two studies [23,24] investigated the use of CAF/AM vs CAF/SCTG. RD and RW were found to have statistically significant difference in favour of test group (CAF+AM). This could be attributed to improved capacity of AM to induce creeping attachment. However, CAF+SCTG showed statistically significant difference in CAL as compared to CAF+AM. SCTG with CAF is the gold standard for treating Miller class I and II gingival recession defects, but subject morbidity is increased due to the existence of a second surgery location. According to Lafzi A et al [23], satisfaction with amniotic membrane aesthetic results was found to be higher than SCTG. In fact, Ghahroudi et al. [24] reported less pain and edema with AM as compared to SCTG. This could be attributed to lack of need of second surgery with AM and due to presence of antimicrobial and anti-inflammatory factor in AM, which include elastase- inhibiting factor and interlukin-1 receptor antagonist (Table 2). Several limitations were observed in this meta- analysis. First was a smaller number of RCTs in the literature. Secondly, no study has evaluated the long term follow up beyond 6 months, except one. This missing data remains a priority for future research. Thirdly, the surgical technique has not been discussed in most of the studies that can alter clinical outcomes. Lastly, the AM used in the studies had been procured from different companies. These different processing methods might have led to deterioration of some of the properties of the material. These methods reduce cellular viability, selective elution of soluble proteins and effects angiogenic factor profile of AM..

## Conclusion:

The use of AM membrane showed comparable results in improving RD, RW, or CAL in the treatment of Miller Class-I and Class-II gingival recession compared to the other treatment modalities such as; PRF, Chorion Membrane, Collagen Membrane and CAF alone. Increase of WKG was found to be significantly more in all the other treatment modalities as compared to AM. However, RD and RW were found to show statistically significant decrease in CAF+AM group than CAF+SCTG, though CAL gain was more with CAF+SCTG. Thus, Amnion membrane seemed to show comparable results with SCTG. Better handling with self-adhering properties, less surgical time, bioavailability, less pain, good aesthetics and presence of growth factors make it better options than SCTG and PRF, where width of keratinized gingiva is adequate.

## Figures and Tables

**Table 1:** Main characteristic of the included study

**Authors/Publicationyear**	**StudydesignandFollowupperiod**	**No.ofpatientsanddefectsites(samplesize)age**	**Treatedteeth,recessiontype**	**Intervent-ion**	**Control**	**Outcomesreported**
GhahroudiAA,2013	RCT-SM6months	N=22;DS=71Age:>18years	Recession:MillerclassIandII	CAF+AM	CAF+SCTG	RD:mean+SD(mm)KTW:mean+SD(mm)RW:mean+SD(mm)CAL:mean+SD(mm)PD:mean+SD(mm)PI:mean+SD(%)BOP:mean+SD(mm)TKG:mean+SD(mm)%RC:mean+SD(%)
ChakraborthyS*etal*.2015	RCT-SM6months	N=12;DS=24Meanage:33.75±6.89years	Recession:MillerclassIandII	CAF+AM	CAF+CM	RD:mean+SD(mm)%RC:mean+SD(%)KTW:mean+SD(mm)RW:mean+SD(mm)CAL:mean+SD(mm)
LafziA*etal*.2016	RCT-SM6months	N=11;DS=30Meanage:34±12years	UptopremolarsinmaxillaandmandibleRecession:MillerclassIandII	CAF+AM	CAF+SCTG	RD:mean+SD(mm)KTW:mean+SD(mm)RW:mean+SD(mm)CAL:mean+SD(mm)PD:mean+SD(mm)GI:mean+SD(%)PI:mean+SD(%)%RC:mean+SD(%)
JainA*etal*.2017	RCT-Paralleldesign6months	N=30;DS=30Age:18-55years	Recession:MillerclassIandII	CAF+AM	CAF+PRF	RD:mean+SD(mm)KTW:mean+SD(mm)PI:mean+SD(%)
IrfanM*etal*,2017	RCT-SM6months	N=10;DS=20Age:18-40yearsMean:37.52years	Recession:MillerclassIandII	CAF+AM	CAF	RD:mean+SD(mm)KTW:mean+SD(mm)RW:mean+SD(mm)CAL:mean+SD(mm)PD:mean+SD(mm)TKG:mean+SD(mm)
GautamA,2017	RCT-SM6months	N=15;DS=20Age:21-52years	MaxillaryandMandibularcanineRecession:MillerclassI	CAF+AM	CAF+PRF	RD:mean+SD(mm)KTW:mean+SD(mm)RW:mean+SD(mm)CAL:mean+SD(mm)PD:mean+SD(mm)%RC:mean+SD(%)
GuptaA*etal*.2018	RCT-SM6months	N=10;DS=20Age:20-50years	Recession:MillerclassIandII	CAF+AM	CAF+CM	RD:mean+SD(mm)CAL:mean+SD(mm)PD:mean+SD(mm)GI:mean+SD(%)PI:mean+SD(%)TKG:mean+SD(mm)
MahajanR*etal*.2018	RCT-SM6months	N=12;DS=24Age:18-40yearsMeanage:29	Recession:MillerclassIandII	CAF+AM	CAF+Collagenmembrane	RD:mean+SD(mm)GI:mean+SD(mm)CAL:mean+SD(mm)PD:mean+SD(mm)PI:mean+SD(%)TKG:mean+SD(mm)%RC:mean+SD(%)
ShalabyHKandMorsySM,2019	RCT-Parallelstudy	N=30;DS=30Age:18-55years	MaxillaryanteriorteethorpremolarsRecession:MillerclassIandII	CAF+AM	CAF+PRF	RD:mean+SD(mm)%RC:mean+SD(%)KTW:mean+SD(mm)CAL:mean+SD(mm)PD:mean+SD(mm)
KumarS*etal*.2020	RCT-SM5years	N=51;DS=102Age:18-40yearsMeanage:35.6years	UpperloweranteriorpremolarareaRecession:MillerclassI	CAF+AM	CAF	RD:mean+SD(mm)KTW:mean+SD(mm)RW:mean+SD(mm)CAL:mean+SD(mm)PD:mean+SD(mm)TKG:mean+SD(mm)%RC:mean+SD(%)
Anamika*etal*.2020	RCT-SM6months	DS=30	MillerclassIandII	CAF+AM	CAF	RD:mean+SD(mm)

**Table 2:** Data related to control and test group considering periodontal parameters at baseline and final evaluation

**Authors/Publicationyear**	**RD(mm)**		**CAL(mm)**		**RW(mm)**		**WKG(mm)**	
	**Baseline**	**6 months**	**Baseline**	**6 months**	**Baseline**	**6 months**	**Baseline**	**6 months**
**CAF+AMv/sCAF+SCTG**
GhahroudiAA(2013)TEST	3.43±1.741	1.13±1.452	4.99±1.403	2.64±1.474	3.89±1.192	1.25±0.496	2.76±1.664	3.44±1.298
CONTROL	4.12±1.986	1.88±1.467	5.98±2.055	3.82±1.593	4.38±0.852	2.93±1.801	2.39±1.277	3.34±1.610
ArdeshirLafzi(2016)TEST	3.13±0.4	1.13±1.26	4.3±0.62	2.16±1.31	4.33±0.84	1.66±1.67	3.13±0.3	3.23±0.32
CONTROL	3.43±0.63	0.8±0.8	4.43±0.9	1.66±0.86	4.5±0.5	2.1±1.04	3.53±1.2	3.53±0.83
**CAF+AMv/sCAF**
KumarS(2020)TEST	2.95±0.89	0.43±0.49	4.40±1.16	1.53±0.52	3.10±0.41	0.49±0.50	3.00±0.75	4.71±0.22
CONTROL	2.70±0.85	0.50±0.45	4.10±0.89	1.64±0.50	3.20±0.79	0.55±0.47	3.10±0.71	4.62±0.25
Anamika(2020)TEST	2.53±0.83	1.90±1.54						
CONTROL	2.60±0.83	1.67±1.18						
MohamedIrfan(2017)TEST	2.9±0.87	0.4±0.51	4.3±1.5	1.4±0.51	3.2±0.42	0.5±0.52	2.9±0.73	4.7±0.67
CONTROL	2.5±0.90	0.3±0.48	3.6±0.84	1.3±0.48	3±0.81	0.40±0.51	3±0.66	4.3±0.67
**CAF+AMv/sCAF+COLLAGENMEMBRANE**
MahajanRetal(2018)TEST	3.17±0.83	0.83±0.80	4.16±0.83	1.70±0.86				
CONTROL	3.08±0.79	1.25±0.83	4.12±0.80	1.90±0.94				
**CAF+AMv/sCAF+CHORIONMEMBRANE**
SonaliChakraborthy(2015)TEST	7.33±1.44	5.75±1.14	9.00±1.86	6.83±1.53	9.00±1.71	6.50±1.51	3.42±0.51	4.42±0.51
CONTROL	7.00±1.86	5.00±1.54	9.331±1.44	7.75±1.22	9.08±1.78	6.92±1.51	3.33±0.49	4.75±0.45
GuptaA(2018)TEST	8.00±1.56	7.00±1.69	9.60±2.22	8.50±2.01				
CONTROL	7.90±1.52	7.10±1.37	9.50±1.50	8.40±1.34				
**CAF+AMv/sCAF+PRF**
Jainetal(2018)TEST	2.800±0.862	1.00±1.00					3.00±0.535	3.667±0.488
CONTROL	2.733±0.799	1.400±0.633					2.733±0.704	3.267±0.594
GautamA(2017)TEST	2.17-0.61	0.12-0.21						
CONTROL	2.10-0.58	0.23-0.27						
**Shalaby(2019)**
TESTMedian	4	1	5	3			3	4
range	2.00-4.00	0.00-2.00	4.00-6.00	1.00-5.00			2.00-4.00	3.00-5.00
CONTROLMedian	3	1	5	3			3	3
range	2.00-4.00	0.00-2.00	4.00-6.00	1.00-5.00			2.00-4.00	3.00-4.00
